# Bimodal and Hysteretic Expression in Mammalian Cells from a Synthetic Gene Circuit

**DOI:** 10.1371/journal.pone.0002372

**Published:** 2008-06-04

**Authors:** Tobias May, Lee Eccleston, Sabrina Herrmann, Hansjörg Hauser, Jorge Goncalves, Dagmar Wirth

**Affiliations:** 1 Department of Gene Regulation and Differentiation, Helmholtz Centre for Infection Research, Braunschweig, Germany; 2 Department of Engineering, University of Cambridge, Cambridge, United Kingdom; Columbia University, United States of America

## Abstract

In order to establish cells and organisms with predictable properties, synthetic biology makes use of controllable, synthetic genetic devices. These devices are used to replace or to interfere with natural pathways. Alternatively, they may be interlinked with endogenous pathways to create artificial networks of higher complexity. While these approaches have been already successful in prokaryotes and lower eukaryotes, the implementation of such synthetic cassettes in mammalian systems and even animals is still a major obstacle. This is mainly due to the lack of methods that reliably and efficiently transduce synthetic modules without compromising their regulation properties.

To pave the way for implementation of synthetic regulation modules in mammalian systems we utilized lentiviral transduction of synthetic modules. A synthetic positive feedback loop, based on the Tetracycline regulation system was implemented in a lentiviral vector system and stably integrated in mammalian cells. This gene regulation circuit yields a bimodal expression response. Based on experimental data a mathematical model based on stochasticity was developed which matched and described the experimental findings. Modelling predicted a hysteretic expression responsewhich was verified experimentally. Thereby supporting the idea that the system is driven by stochasticity.

The results presented here highlight that the combination of three independent tools/methodologies facilitate the reliable installation of synthetic gene circuits with predictable expression characteristics in mammalian cells and organisms.

## Introduction

To date, most of the synthetic gene networks have been created for bacteria or for lower eukaryotes [1]–[7]. They were successfully employed to investigate different regulatory networks within these biological systems. A wealth of information could be provided by these studies. To facilitate systems biology based approaches for the elucidation of complex regulatory networks involved in immunology, developmental biology or infection, the development of appropriate synthetic modules as well as their establishment in mammalian cells and animals is indispensable. A thorough understanding and mathematical description of these modules in complex organisms would further pave the way to exploit such circuits within systems biology and also for applications in gene or cell therapy [8].

Pioneering studies demonstrated that the creation of synthetic gene circuits is feasible also in mammalian systems. The designed devices encompassed a toggle switch [9], a hysteretic switch [10] and a time-delay circuit [11]. A major limitation is that the manipulation of mammalian cells is tedious and time-consuming. Especially stable integration of synthetic gene circuits remains a challenge and is up to now only achieved in immortalized cell lines which usually do not or only partially reflect relevant properties of the cells they have been derived of. This can be partially attributed to the genetic changes which are required to provide unlimited cell growth. To implement synthetic cassettes in primary cells novel tools have to be developed.

Viruses have evolved strategies to efficiently transduce their genetic information to mammalian cells. In particular, adenoviruses, adeno-associated viruses and retroviruses were exploited for their potential to genetically modify mammalian cells [12], For this purpose, non-replicating viral vectors have been developed that can be transduced with the help of packaging cells [e.g. 13], [14]. While non-replicating extrachromosomal vectors (e.g. derived from adenoviruses) remain in an extrachromosomal state they lead to a transient expression in proliferating cells. Retroviral and especially lentiviral vectors are the optimal tools to provide stable integration of expression cassettes into the host's genome and thus guarantee the maintenance of the cassettes in replicating cells. Pseudotyping of lentiviral vectors allow to to transduce a broad range of different cell types from different species. Importantly, they are also capable of infecting slowly or non-proliferating cells and thus are preferential tools for many primary cells including stem cells.

Gene expression patterns in eukaryotes can be classified into two distinct types: graded and bimodal [15]. In the graded pattern, cells respond uniformly to increasing levels of the stimulant. In contrast, in the bimodal pattern, the level of the stimulant affects only the probability that a gene will be expressed in a given cell; this expression is always “maximal”. Bistable expression of metabolic genes such as those for galactose utilization in yeast [16] and lactose utilization in E.coli [17] have been shown to result from positive feedback. One prominent example for bistability in eukaryotes is the cell-cycle oscillator, a biochemical system that has been shown to promote irreversible transitions between distinct mitotic and interphase states [18]–[20]. But also in the immune system the T helper activation is governed by an bimodal expression of the transcription factor NFATc2 which integrates graded T cell receptor activation into a bimodal IL2 expression [21], [22].

The bimodal expression mode may lead to multimodal variation in the phenotype of an isogenic cellular culture and in organisms (i.e. differentiation). In genetic networks this confers a memory to past events or environmental changes. Thus, stochasticity is an important feature of many biological processes in mammalian cells. Several means have been described to achieve a bimodal expression pattern. Amongst them are positive feedback loops and mutual repression [23]. As an example, the latter can be accomplished using two interlinked negative feedback loops which create a toggle switch [4], [9]. This toggle switch setup was employed in another study as a memory subsystem. This was implemented and used for recording the outputs of a larger DNA-damage sensor network [24]. Bimodal expression was further achieved by employing two opposing transcriptional regulators [25]. Becskei et al. (2001) [26] finally proved that in yeast even a less complex network based on the tet-system can result in a bimodal expression pattern upon gradual increase of the inducer tetracycline.

The aim of this study was to provide and evaluate a strategy to efficiently implement a bimodal synthetic gene circuit in mammalian cells. For this purpose, three different methodologies were exploited. A synthetic positive feedback system resulting in binary expression response was transduced via lentiviral transduction. This combination ensures that the synthetic gene circuit can be installed a broad range of mammalian cells including primary cells. After stable integration into the host cell the synthetic gene circuit displayed a bimodal expression pattern and the resulting experimental data were used to construct a mathematical model. The model followed closely the observed in vivo data and predicted a strong hysteretic response of the positive feedback loop. The in silico data were experimentally verified demonstrating the value of this concerted approach.

## Results

### Bimodal expression from a lentiviral transduced positive feedback loop

Efficient transduction and long-term stable expression of transgenes is achieved with the help of lentiviral transfer vectors both for basic research and gene therapeutic approaches [27]. Several generations of lentiviral vectors have been developed and allowed to successively reduce viral sequences [28]. These modifications led to higher safety. Importantly, through the deletion of the viral cis-acting regulatory elements (viral promoter, enhancer) the expression pattern is determined by the nature of the transduced expression cassette.

**Figure 1:**
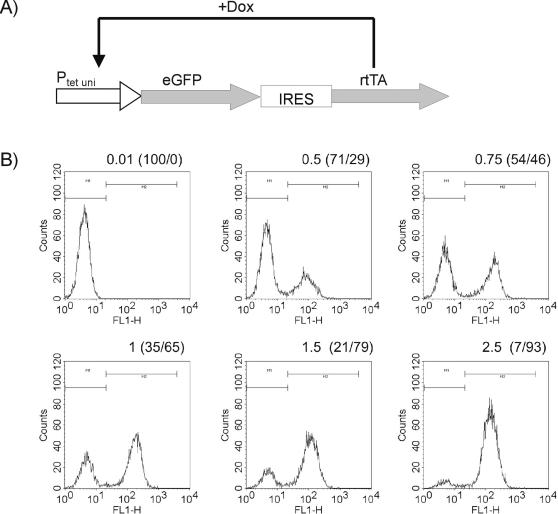
Setup and expression pattern of the positive feedback loop. (a) Vector map of pJSARGFP transducing the positive feedback loop. (b) NIH3T3 cells were stably transduced through lentiviral infection with pJSARGFP. A representative clone was cultivated for four days with the indicated Dox concentrations (in µg/ml) and analyzed for GFP expression using flow cytometry. The numbers in brackets represent the percentage of cells which fall in the respective marker regions. The bimodal response is clearly visible from this data.

We chose a 3^rd^ generation lentiviral vector devoid of any viral promoter elements for transduction of the synthetic expression module. This excluded any interference in between the viral vector backbone and the synthetic gene regulation circuit. A transcriptionally regulated gene circuit based on the Tetracycline(tet)-system was implemented in the lentiviral vector. This synthetic module was designed to generate a positive feedback response. Initiation of the positive feedback loop is achieved by a low basal level of the transactivator rtTA (Fig. 1A). This configuration meets the requirements for a synthetic gene circuit as its regulation is simple and homogenously achievable without any adverse/side effects mediated by the inducer. Secondly, it facilitates rapid induction and repression kinetics as well as multiple cycles of induction and repression. To precisely follow the induction/repression properties of this gene circuit a destabilized enhanced green fluorescent protein (eGFP) mutant [29] is coregulated, thereby reflecting the transcription of the transactivator. eGFP can be monitored in viable cells and facilitates the monitoring of expression on single cell level e.g. by flow cytometry.

**Figure 2:**
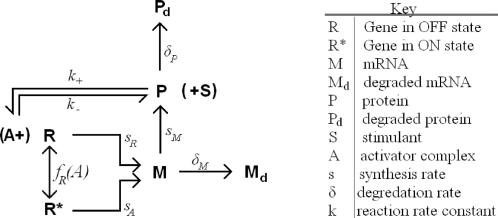
Reaction scheme for the feedback system. Each node corresponds to a reaction governed by a transition propensity, with lines and arrows representing reactants and products, respectively. A dotted line signifies that the ‘reactant’ is not consumed.

**Figure 3:**
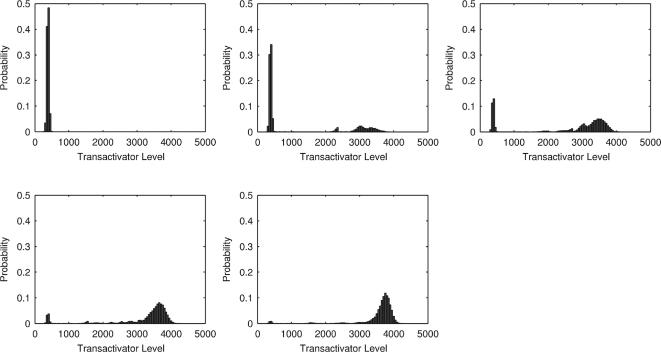
Simulations of the expression behaviour of the positive feedback loop. The simulations based on the Gillespie algorithm were run as described in the text. Increasing concentrations of Dox (0, 0.5, 1, 1.5 and 2.5 µg/mL) were applied as indicated. The resulting transactivator molecule counts were monitored. The population histograms show the probability of a respective transactivator level. The system parameters were k_on_ = 20, k_off_ = 20, sA = 50, sR = 5, sP = 0.2, δ_M_ = 0.1, δ_P_ = 0.05, k+ = 20 and k− = 10.

**Figure 4:**
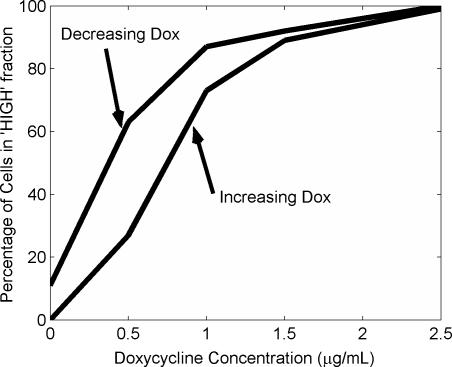
Computer simulations predict hysteretic behaviour. Based on the simulations in Figure 3 and corresponding simulations of the on→off transitions. The lower curve represents the percentage of cells in the ‘HIGH’ expression region as the concentration of Dox is gradually increased. The upper curve shows the percentage of cells in the ‘HIGH’ expression region after a transition from a saturating Dox level to that shown.

**Figure 5:**
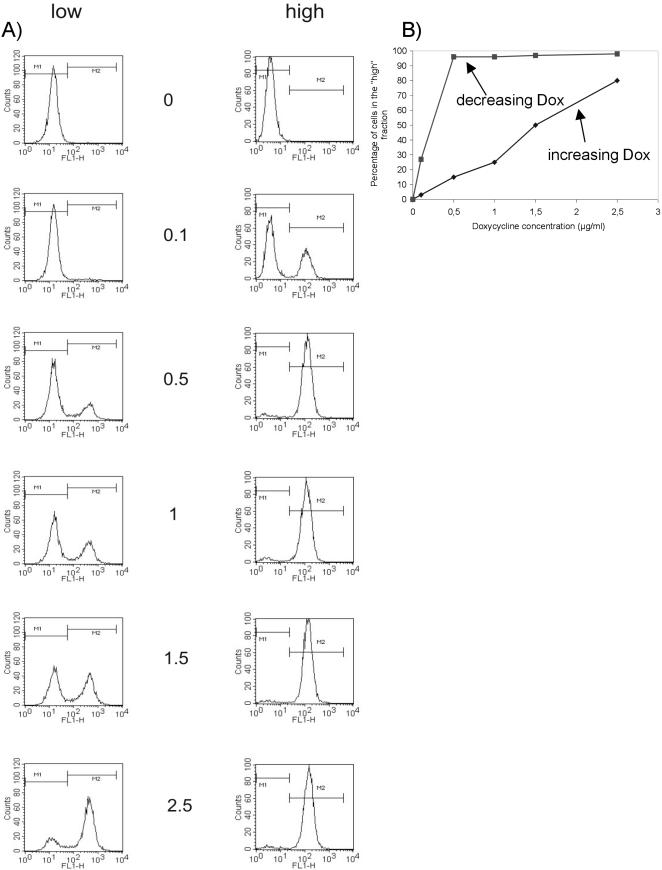
Hysteretic behaviour of cells carrying the positive feedback loop. (a) Cells carrying the autoregulated cassette (Fig. 1A) were cultivated with 1 µg/mL of Dox for 4 days and then sorted. The eGFP positive cell population is designated with ‘high’, the eGFP negative cell population is designated with ‘low’. After sorting the cells were cultivated for additional 4 days with the indicated Dox concentrations. (b) The number of GFP positive cells is plotted against the applied Dox concentration. The blue line reflects the off→on experiment, the red line the corresponding on→off experiment.

We implemented the autoregulatory expression module in NIH3T3 cells using lentiviral gene transfer. Cell clones were established and expression was monitored on the single cell level by flow cytometry (Data from a representative clone is shown.). Increasing concentrations of the inducer were applied to investigate the pattern of expression response (Fig. 1B). Saturating inducer concentrations (2.5 µg/mL Doxycycline (Dox)) caused homogeneous activation and all cells showed eGFP expression. At non-saturating concentrations of the inducer (0.5–1.5 µg/mL) the population was split and yielded a binary population distribution. By varying the inducer concentration, the ratio of expressing to non-expressing cells could be adjusted. This is a specific property of the positive feedback loop since constant high level expression of the transactivator yielded a graded transcriptional response (data not shown and [30]). Further, constitutive co-expression of the transactivator in these cells turned the response to a graded mode [data not shown].

### Mathematical modelling of the binary expression module

A mathematical description of the synthetic module was produced. Standard stochastic techniques were employed to try and recreate the binary response [31], [32]. For this purpose, the particular regulatory network was modelled as a discrete, multi-valued, continuous time Markov process. This mathematical simulation was based on the reaction scheme proposed in Fig. 2. For the sake of simplicity, the eGFP loop that is simultaneously activated and that mirrors tTA expression was excluded. In this reaction scheme, the ‘allowable’ events are synthesis and degradation of mRNA (at two rates, ON and OFF, respectively), synthesis and degradation of proteins, the binding of Dox to the rtTA to form the activator complex, gene switching upon binding of the activator complex to the promoter and a term which reflects competition for Dox within the cellular population. The system was simulated using the Gillespie algorithm [32]. Since exact values for the state transition propensities are not available, we used typical values from Kaern et al. (2005)[33], which were then uniformly scaled to calibrate with the expected time scales.

The simulations were run using different initial concentrations of the inducer Dox (off→on simulation). Fig. 3 summarises the results obtained from 20,000 independent simulations per given Dox concentration. These results demonstrate two distinct steady state levels of the transactivator, a ‘HIGH’ and a ‘LOW’ fraction. With increasing concentration of Dox the probability for the ‘HIGH’ fraction increases. Thus, the simulation reflects the bimodal distribution across the population as observed in the *in vivo* setting.

### Hysteretic response of the positive-feedback

Strong positive feedback loops are often associated with hysteresis [34]. We tested if the mathematical algorithms as applied here would predict such behaviour; for this purpose, the simulations were run with inducer concentrations of 0, 0.5, 1.0, 1.5 and 2.5 µg/mL, but applying initial condition vectors that were taken from the end of the earlier simulations at saturating inducer levels (on→off simulation). The mathematical simulation showed that for the on→off situation a much lower concentration of the inducer was needed to switch off the system and decrease the probability of a given cell being in the ‘HIGH’ fraction. A summary of the on→off simulations is provided in Figure 4; for comparison, the corresponding graph for the off→on simulation is also indicated. Thus, the history of the system clearly defines the distribution within the population: for example, while a shift from 0 to 1 µg/mL of Dox gives rise to a clear binary pattern with 70% of cells in the ‘HIGH’ fraction, the downward shift from 2.5 µg/mL to 1 µg/mL results in more than 80% of the cells remaining ‘HIGH’. Importantly, for all applied concentrations of Dox, the final distribution favoured the ‘HIGH’ expression level for the on→off simulation.

We asked if this mathematical prediction is supported by experimental data and transferred the conditions used for simulation to isogenic cells with different cultivation histories. For this purpose, cells were cultivated for four days with non-saturating inducer concentrations (1 µg/ml). This treatment generates two populations (see Fig. 1B). Both cell populations were sorted and subsequently again cultivated with different inducer concentrations (0, 0,1, 0,5, 0,75, 1,0, 1,5, 2,0 and 2,5 µg/mL Dox). As expected, both populations behaved uniformly for 0 Dox and 2,5 µg/mL Dox: both ceased expression of eGFP upon cultivation without any Dox, and showed fully activated expression under saturating conditions (2.5 µg/mL). However, the cell pools displayed an inhomogeneous expression pattern at non-saturating levels of 0.1–2.0 µg/mL: while cells from the ‘low’ population provided a bimodal distribution, the ‘high’ population cells remained fully induced at inducer levels of 0.5 µg/mL and above (Fig. 5A). Only cells, cultivated with low concentrations of the inducer (0.1 µg/mL and below) also showed a bimodal expression pattern resulting in two populations. In (Fig. 5B) the obtained expression are plotted against the Dox concentration. Together, these data confirm the hysteretic behaviour of the cells as predicted from the mathematical model.

## Discussion

A goal of synthetic biology is to describe and understand naturally occurring networks by constructing synthetic analogues. Mathematical modelling of such synthetic modules allows to describe and evaluate the installed networks. Further, predictions can be made which are subsequently experimentally verified. However, the installation of synthetic gene circuits in cells of higher eukaryotes is still not sufficiently solved. In particular, primary cells or even animals are so far not available for systems biology approaches. This is attributed not only to the genetic and phenotypic complexity of these natural systems but also to the lack of reliable methods to implement synthetic cassettes. Here, we show that expression cassettes conferring bimodal expression can be efficiently transduced with the help of lentiviral vectors. The results presented here were obtained with an immortalized cell line (NiH3T3 cells). However, the real strength of lentiviral transduction is that not only cell lines but also primary cells of different origin and from different species can be transduced. Preliminary data demonstrate that transduction of the synthetic gene regulation circuit is feasible and efficient also in primary cells, Importantly, the expression characteristics of the positive feedback loop are comparable to those observed in the NiH3T3 cells (data not shown). Establishing a mathematical model based on stochasticity we further describe and predict expression patterns.

Stochasticity is frequently encountered in biological systems. In particular, they contribute to transcriptional and translational processes [2], [35]–[38]. Generally, it would be expected that stochasticity would compromise the precise control of gene expression levels eventually with detrimental consequences. Indeed, this has been described for several situations [1], [16], [39]. However, stochasticity also provides a mechanism to generate certain diversification of individual cells within a population–and thus is advantageous under certain conditions, e.g. when the cell encounters stress conditions [40]. Different natural networks in which stochastic expression leads to a phenotypic heterogeneity of isogenic cells have been described [reviewed in 33]. This includes the lysogeny decision in phage lambda; here, an initially homogenous population is split due to molecular fluctuation [41]. Also, the HIV-1 Tat expression module gives rise to fluctuations that create distinct phenotypes from a single proviral integration [42].

In mammalian systems, stochasticity seems to play a particularly important role in development and differentiation processes such as hematopoiesis [43]. Such decisions are also encountered in networks governing apoptosis [44] and the cell cycle [34]. Also, stochasticity can be involved in diseases, in particular in those which arise from transcription factor haploinsufficiency; upon stochastic simulation evidence was given that haploinsufficient systems are more susceptible to disturbances in expression [45]. Experimental evidence confirmed this result, demonstrating a decreased probability of gene activation in haploinsufficient cells [46]. In summary, it seems that stochastic fluctuations, whilst problematic in a homeostatic context, may prove to be essential within the context of development and differentiation of cellular populations.

In previous studies it was demonstrated that a bimodal expression pattern was realized when a repressor and a transactivator compete for the same DNA binding site [25] by two interlinked negative feedback loops [4], [9] or by positive feedback and mutual expression [23]. Here, we show that a less complex network consisting of a transactivator and its responsive promoter is sufficient for provoking bimodal expression in a mammalian system. In this respect, the described positive feedback modules represent ideal building blocks to artificially provoke all-or-nothing-answers in mammalian cells.

The mathematical model predicts a hysteretic expression pattern from the synthetic positive feedback loop. This prediction was experimentally verified. Such a strong a strong hysteretic response is expected to occur only from strong positive feedback loops. The hysteretic response of feedback modules can be tuned by the strength of the positive feedback [34]. To achieve such a response with the synthetic modules presented here, transactivator mutants eliciting activation at different inducer levels [47] might be of benefit. Furthermore, the hysteretic response can be modulated to different strengths through the connection of a positive feedback loop with a transcriptional repressor [10].

This latter approach pinpoints that for the establishment of complex multigene networks, several non-interacting switches (transactivators) or relais (promoters) are needed. Progress has been made in the creation of independent gene regulation for mammalian systems [for review see 48]. The combination of different systems within single cells or animals would allow to differentially control distinct circuits. As exemplified by this study the lentiviral transduction of a synthetic positive feedback circuit proves useful. Exploiting this tool for transduction of independently regulated synthetic modules will allow to reliably install more complex networks in mammalian cells including primary cells. Thereby, it provides an important step to synthetically modify these systems for systems biology approaches.

## Materials and Methods

### Cell culture

Murine fibroblast NIH3T3 cells (ATCC CRL-1658) and 293T cells (human embryonal kidney cells transduced with the simian virus 40 large T antigen) were maintained in Dulbecco's modified Eagle's medium (DMEM). Media were supplemented with 10% fetal calf serum, 2 mM L-glutamine and penicillin (10 U/ml), and streptomycin (100 µg/mL). Doxycycline (Dox) was dissolved in ethanol, filtered and stored at −20°C. The genetic insert, pJSARGFP (synonym: TREAutoR3) was described previously [49].

### Gene transduction

Lentiviral production was achieved through calcium phosphate transfection of four plasmids. For this purpose, 2×10^5^ (293T) cells were plated per 9 cm^2^ one day prior to transfection. The lentiviral expression module was transfected into 2×10^5^ 293T cells, along with the helper plasmids pLP1 (gag-pol), pLP2 (Rev) and pLP/VSV-g (VSV g) (all obtained from Invitrogen). For transfection, 1,3 µg of lentiviral expression plasmid with 0,9 µg of pLP1, 0,3 µg of LP2, and 0,5 µg of pLP/VSVg were mixed with 15 µL 2.5 M CaCl_2_, and adjusted with H_2_O to give a final volume of 150 µL. The DNA solution was dropwise added to 150 µL HEBS (280 mM NaCl, 50 mM HEPES, 1.5 mM Na_2_HPO_4_, pH 7.1) under vigorous vortexing and incubated at room temperature for 10 min. After 12 h the transfection media was renewed and the virus containing supernatant was collected two and three days after transfection. The supernatant was filtered (pore size 0.45 µm) and frozen at −70°C. For infection 1*10^5^ NIH3T3 were plated per 9 cm^2^ dish a day prior to infection. At the day of infection the viral supernatant was supplemented with Polybrene (8 µg/mL) and added to the cells for 8 h. The multiplicity of infection was set to 1 to obtain single copy integration of the synthetic gene circuit.

### Reporter Gene Expression

To determine eGFP expression 1×10^5^ cells were cultivated per 6 well plate with the indicated Dox concentrations for the indicated time spans. The cells were washed with PBS, trypsinized and centrifuged at 200 g for five minutes. Cells were stained with Propidium iodide (50 µg/mL) to exclude dead cells from the analysis. The cells were analysed for eGFP expression by flow cytometry using a FACSCalibur (Becton Dickinson).

### Mathematical modelling of the binary expression module

A mathematical description of the synthetic module was developed. This mathematical simulation was based on the reaction scheme proposed in Fig. 2. The particular regulatory network was modelled as a discrete, multi-valued, continuous time Markov process which is amenable to standard stochastic techniques. The actual simulations were performed using the Gillespie algorithm as implemented in the SimBiology package, produced by The MathWorks. [31], [32]. In essence, the Gillespie algorithm maintains a count of the number of molecules of each species currently in the cell. These counts are updated at random times based on a set of propensities which are defined and calculated using a given reaction scheme.
